# Mitochondrion-to-nucleus communication mediated by RNA export: a survey of potential mechanisms and players across eukaryotes

**DOI:** 10.1098/rsbl.2024.0147

**Published:** 2024-07-10

**Authors:** Giorgio Muneretto, Federico Plazzi, Marco Passamonti

**Affiliations:** ^1^ Department of Biological, Geological and Environmental Sciences, University of Bologna, Bologna, Italy

**Keywords:** mitochondrial retrograde response, mito-nuclear interactions, regulatory RNAs, small mitochondrial highly transcribed RNAs, mitochondrial export

## Abstract

The nucleus interacts with the other organelles to perform essential functions of the eukaryotic cell. Mitochondria have their own genome and communicate back to the nucleus in what is known as mitochondrial retrograde response. Information is transferred to the nucleus in many ways, leading to wide-ranging changes in nuclear gene expression and culminating with changes in metabolic, regulatory or stress-related pathways. RNAs are emerging molecules involved in this signalling. RNAs encode precise information and are involved in highly target-specific signalling, through a wide range of processes known as RNA interference. RNA-mediated mitochondrial retrograde response requires these molecules to exit the mitochondrion, a process that is still mostly unknown. We suggest that the proteins/complexes translocases of the inner membrane, polynucleotide phosphorylase, mitochondrial permeability transition pore, and the subunits of oxidative phosphorylation complexes may be responsible for RNA export.

## 1. Mitochondrial retrograde response

Mitochondria communicate with the nucleus to coordinate various cellular processes and to maintain cellular homeostasis. Mitochondrial retrograde response (MRR), defined as the cellular response to changes in the mitochondrial functional state, is already an overall well-studied process [1]. Reactive oxygen species (ROS), Ca^2+^ and acetyl-CoA are some examples of molecules that exit the mitochondrion as signals to the nucleus, leading to up- or downregulation of nuclear genes [2,3]. For example, the action of ghrelin, the hunger-promoting peripheral hormone, on human neuronal cells that present ghrelin receptors causes a cellular cascade leading to the generation of ROS in mitochondria [2]. The production of ROS promotes uncoupling protein 2 gene transcription in the nucleus, leading to reduced ROS production, increased mitochondrial activity and, lastly, neuronal activity-dependent synaptic plasticity [2].

Differently from other molecules, RNAs encode precise information and can influence gene expression in multiple ways. RNA interference (RNAi) is the sequence-specific suppression of gene expression through transcriptional or post-transcriptional gene silencing, mediated by an RNA molecule [4]. In the latter case, the RNA forms a double strand with the target messenger (mRNA), leading to its degradation. An example of RNAi is given by the activity of microRNAs (miRNAs), a class of small non-coding RNAs that work in this way. Most commonly, nuclear miRNA genes are transcribed as pri-miRNAs and matured by the microprocessor complex into pre-miRNAs about 70 bases long [5]. The pre-miRNA folds into a hairpin and is exported to the cytoplasm, where it is further processed by Dicer at the looped end and loaded on Ago2, becoming part of the RNA-induced silencing complex that is responsible for the cleavage of the target mRNA [5].

RNAi is found in all major branches of eukaryotes and appears to have evolved multiple times in eukaryotic evolution [4]. Mitochondria produce mitochondrially encoded small RNAs [6], which have been hypothesized to be involved in MRR [7]. Further evidence of RNA-mediated MRR came from the study of other transcripts, including small mitochondrial highly transcribed RNAs (smithRNAs) [8,9] and mitochondrial transfer RNA (tRNA) fragments (mt-tRFs) [10] (table 1).

**Table 1.** RNAs and mtDNA fragments known to exit the mitochondrion. (RNAs were only detected *in silico* in organisms marked with question marks (’?’).)

nucleic acid	organism	location	pathway	references
smithRNAs	*R. philippinarum*, mouse (?), zebrafish (?), fly (?)	cytoplasm	RNAi	[8,9]
tRNA^Met^	human	cytoplasm	RNAi (?)	[11]
SncmtRNA/ASncmtRNAs	human, mouse	nucleus, cytoplasm	nuclear gene regulation, cell proliferation	[12–16]
mt-tRFs	human, mouse, yeast	cytoplasm	multiple functions	[10]
mt-internal-tRF Glu(UUC)	human	cytoplasm	downregulates the expression of MPC1	[17]
TERC−53	human	cytoplasm	interferes with GAPDH nuclear translocation	[18,19]
mtDNA fragments	human, mouse, yeast	cytoplasm	cellular stress response	[20,21]
mt-caRNAs	human	nucleus, cytoplasm	cellular stress response	[22]

## 2. The case of doubly uniparental inheritance

Although signal-mediated cooperation between mitochondria and nucleus is crucial for the cell, genomic conflicts are also possible between and within the two compartments, each replicating independently and being subject to different levels of selection. Uniparental inheritance of mitochondria is found in most animals as maternal inheritance and is thought to have arisen as a mechanism to reduce inter-mitochondrial conflicts, increasing homoplasmy [23]. In turn, this led to the emergence of additional mito-nuclear sex-related conflicts, like sex determination.

In most animals, mitochondria are transmitted through the egg; male mitochondria are degraded during spermatogenesis or after fertilization [23]. There is a significant exception to uniparental inheritance in metazoans, which is called doubly uniparental inheritance (DUI), that has been found in more than a hundred species of bivalves belonging to six different orders [24–26]. DUI involves the transmission of two sex-specific mitochondrial DNAs (mtDNAs): normally, female mtDNA (F-mtDNA) is transmitted by only females, while male mtDNA (M-mtDNA) by only males. However, males tend to be somatically heteroplasmic for both mtDNAs, while females possess only F-mtDNA [27–30].

DUI has been suggested to evolve as a response to the insertion of selfish elements of putative viral origin that acted as sex distorters. Traces of these elements are visible today in DUI-linked mitochondrial open reading frames specific to the M and F lineages: some of them have been found to be transcriptionally active in DUI germlines [31]. Therefore, it has been suggested that DUI resolves the conflict between the mitochondrial and nuclear genome by segregating them in either germline [31,32]. The two mtDNAs evolved sex-linked features [27,32], including the capacity to affect the sex ratio to increase their chances of being transmitted. This also means that the two genomes may have evolved pathways to affect germline differentiation by regulating nuclear gene expression. This is reflected by the different characteristics of the two mtDNAs, such as different lengths, gene insertions, duplications and deletions, and sex-specific transcripts such as smithRNAs (e.g. [33]).

## 3. Observations supporting retrograde RNA-based signalling machinery

Small non-coding RNAs (sncRNAs) were demonstrated to mediate MRR in the DUI species, *Ruditapes philippinarum*. SmithRNAs are encoded in the mitochondrial genome of *R. philippinarum* and are predicted to target nuclear loci [8]. The functionality of two smithRNAs, M_smithRNA106t and 145 t, has been demonstrated, as well as the existence of selective constraints on several of the putative loci [9]. M_smithRNA106t injected in clams led to a significant reduction of histone H3 methylation levels, while injected M_smithRNA145t led to significantly increased histone H3 acetylation. In the DUI species *Potamilus streckersoni* (Unionidae), it was found that a sncRNA mapping on M-mtDNA may target and silence a nuclear gene upregulated in females [34]. Putative smithRNAs were also identified in gonad samples of three different model species: fruitfly, zebrafish and mouse [9].

Besides smithRNAs, the presence of mitochondrial RNAs outside the mitochondrion has already been documented multiple times (see table 1 for a more extensive list). Four mitochondrially encoded tRNAs were found in the cytosol of the human 293 T-cell line. Interestingly, mt-tRNA^Met^ immunoprecipitated with Ago2 [11], a protein involved in miRNA-mediated silencing [35].

A recent study by Sriram and colleagues [22] uncovered many chromatin-associated RNAs of mitochondrial origin (mt-caRNAs) attached to the nuclear genome in humans, predominantly in gene promoters. Depletion and induction of specific mt-caRNAs altered transcription levels of nuclear genes; knockdown of a group of mt-caRNAs called Sense (i.e. H-strand mapping). Non-coding mitochondrial RNAs (SncmtRNAs) led to a strong suppression of anti-viral and type 1 interferon response pathways. This change was correlated with altered nuclear, and not mitochondrial, gene expression. Therefore, mt-caRNAs are an example of RNA-mediated MRR, acting through transcriptional regulation.

The first sense and antisense (i.e. L-strand mapping) non-coding RNAs (SncmtRNAs and ASncmtRNAs, respectively) of mitochondrial origin, later classified as mt-caRNAs, were long non-coding RNAs characterized in normal human cells [12,13,22]. They were found to be associated with chromatin (preferentially heterochromatin) in the nucleus; moreover, sense RNA expression was strongly correlated with cell proliferation [12]. Multiple studies focusing on the antisense RNAs revealed their tumourigenic properties and explored them as targets for cancer therapy [14–16]. For example, Lobos-González and colleagues demonstrated that tumourigenesis of murine breast cancer was reduced upon knockdown of an ASncmtRNA by inhibiting cell proliferation and inducing apoptosis [16].

Like their nuclear counterparts [36], mt-tRFs are produced by the activity of endo/exoribonucleases, found either in the cytoplasm, suggesting a cytoplasmic origin from exported mitochondrial tRNAs, or in the mitochondrion itself, in which case the mt-tRFs could either translocate to the cytosol or locate and exert their function in the matrix [10]. Each tRF class has been linked to a specific biological function, and several mt-tRFs have been hypothesized to act as miRNAs [10]. One of these is processed in human cells by Dicer, either in the matrix or in the cytoplasm, and later loaded onto Ago2 in the respective compartment [17], a biogenesis similar to miRNAs. This tRF downregulates the expression of mitochondrial pyruvate carrier 1 (MPC1), most likely through interaction with the MPC1 mRNA in the cytosol [17].

To regulate nuclear gene expression, some mitochondrial sncRNAs could access the cytosol at the level of the chromatoid body, which includes extruded mitochondrial cristae and matrix [37]. However, this would constitute a very limited circumstance. Because smithRNAs and other mitochondrial sncRNA activity do constitute a widespread mito-nuclear crosstalk in eukaryotes, understanding how mitochondrial RNAs can be exported to the cytoplasm is of outstanding interest. This knowledge will also shed light on the MRR of the proto-mitochondrion engaged during early eukaryogenesis.

## 4. Ways to deliver functions to the cytoplasm

**Figure 1.**
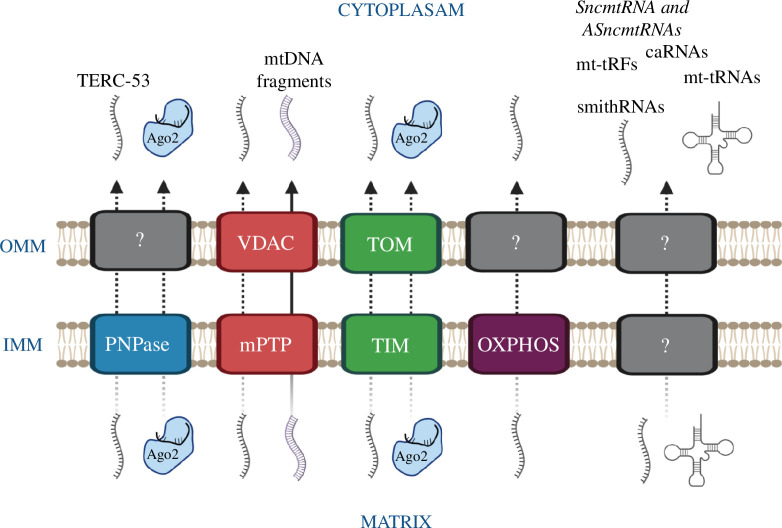
Mechanisms of RNA export from the mitochondrion. Dotted arrows, suggested mechanisms for RNA export from the mitochondrial matrix to the cytoplasm; solid arrow, proved exit routes through mPTP and VDAC. Grey complexes represent unknown carriers/pathways. Molecules engaged in translocation include free RNAs, RNA–Ago2 complexes, DNA fragments, tRNAs and mt-caRNAs. Created with BioRender.com.

The inner mitochondrial membrane (IMM) is less permeable than the outer mitochondrial membrane (OMM). This is mostly owing to the large number of voltage-dependent anion channels (VDACs), which are found in all eukaryotes so far [38]. VDACs are present on the OMM and allow the diffusion of molecules ranging from small metabolites to mitochondrial DNA [39]. On the other hand, translocation across the IMM occurs thanks to a wider number of translocation mechanisms. Moreover, the mitochondrial membrane potential (ΔΨm) is positive on the outer side of the IMM [40], which would favour export over the import of negatively charged RNA molecules. Some of the complexes involved in the import–export of molecules are good candidates for RNA translocation towards the cytosol, namely, we would like to focus attention on translocases of the inner membrane (TIM), polynucleotide phosphorylase (PNPase), mitochondrial permeability transition pore (mPTP) and exapted oxidative phosphorylation (OXPHOS) subunits (figure 1).

### (a) Translocases of the inner membrane

A translocase of the outer membrane (TOM) is present on the OMM. The TIM are protein complexes TIM22 and TIM23, as they have been studied in *Saccharomyces cerevisiae* and other eukaryotes: the TIM23 complex allows the import of presequence-containing proteins; the TIM22 complex mediates the insertion of carrier proteins within the inner membrane [41]. The TOM–TIM supercomplex has been implicated in the import of tRNA^Lys^ into *S. cerevisiae* mitochondria [42], one of the two nuclear tRNAs imported in *S. cerevisiae*, together with tRNA^Gln^ [43]. Notably, tRNA^Lys^ is probably imported in a conformation different from the classical L-shape by binding to the precursor of mitochondrial lysyl-tRNA synthetase and with the contribution of the second isoform of the glycolytic enzyme enolase [42], while tRNA^Gln^ import does not require cytosolic factors [43]. Moreover, tRNA^Lys^ import can be achieved even by crossing the outer membrane through VDAC instead of TOM [42].

The mtDNA of *Trypanosoma brucei* is devoid of tRNA genes, meaning that all tRNAs for mitochondrial use are imported; a similar implication of TOM–TIM supercomplex has been found [44]. The *T. brucei* TOM complex is called atypical TOM (ATOM), owing to fundamental differences in protein composition when compared with the same complex in *S. cerevisiae* [45], while retaining the core components Tom40/ATOM40 and Tom22/ATOM14. TIM23 and TIM22 are not found together; there is a singular integral membrane protein Tim22/TbTim17, which is accompanied by three other core proteins [44].

TIM22/TIM23-mediated RNA export is highly speculative, since no evidence of the process has been found yet. However, in the absence of a known mechanism, it stands as a reasonable hypothesis. RNA export could happen either with the free molecule, similar to tRNA import in *T. brucei* [44], or by binding to another protein, like tRNA^Lys^ import in *S. cerevisiae* [42]. In the latter case, a possible protein involved is Ago2, which would bind a miRNA inside the mitochondrion before being translocated, as hypothesized by Srinivasan & Das [46].

### (b) Polynucleotide phosphorylase

PNPase is a 3′-to-5′ exoribonuclease/poly-A polymerase located in the matrix and in the intermembrane space, specifically as an IMM-bound peripheral protein [47]. In eukaryotes, it appears to be highly conserved, but it is absent in some fungi and in *T. brucei* [47]. Structural studies on the human IMM-bound PNPase led to the proposal of a trimeric protein complex structure with a central channel containing a single-stranded RNA-binding catalytic site [48]. Hairpin structures with a short 3′ overhang are not degraded and interact with the RNA-binding domains for translocation. The ribozyme ribonuclease P (*RNAseP*) cleaves off a precursor sequence on tRNA molecules and, along with the human RNA component of mitochondrial RNA processing endoribonuclease (*RMRP*), was specifically bound to PNPase, which was functional for their import, by means of a hairpin loop [49]. By truncating different RNA portions, the import signal was restricted to a 103–140 nt long region of *RnaseP,* containing a hairpin structure similar to a second loop found in RMRP [49]. Notably, by fusing a normally non-imported RNA with either of the two loop sequences at the 5′ end, the RNA could be imported into mitochondria [49]. Furthermore, PNPase participates in the translocation of a miRNA into the matrix in humans and mice [50].

Human telomerase RNA component (*hTERC*) is a long, non-coding RNA that serves as the template of the telomeric repeat. It enters the mitochondrion, where it is processed into *hTERC-53* to a final length of 195 nt [18,19]. It is known that *hTERC* has a loop with a similar sequence/structure to the hairpin that is involved in PNPase-mediated import [18]. Interestingly, deletion of this region significantly increased *hTERC* import, and the first 52 nucleotides were shown to be necessary.

Borowski and colleagues [51] showed that the human RNA degradosome (mtEXO) is composed of PNPase and Suv3. A possible role for mtEXO is the prevention of double-stranded RNA (dsRNA) formation in the matrix through RNA degradation. Hypomorphic mutations in *pnpt1* (encoding for the PNPase monomer) lead to the cytoplasmic accumulation of mitochondrial dsRNAs [52]. This finding supports the role of the matrix-located PNPase not only in degrading dsRNA, as part of mtEXO, but also in preventing dsRNA from escaping to the cytosol. In another study, PNPase overexpression led to increased *hTERC* import and an accumulation of cytosolic *hTERC-53* [18]. The presence of cytosol determined the export of most of the mitochondrial *hTERC-53* with or without PNPase overexpression, suggesting that cytosolic factors are required for the process. These results could originate from the export being a more efficient process than the import, as the authors suggested, but the role of PNPase in *hTERC-53* export is not excluded.

In *R. philippinarum*, pre-smithRNAs are predicted to fold into a secondary structure mostly lacking a 3′ overhang [8]. In case of PNPase interaction, this would avoid degradation and favour translocation [48]. PNPase has been co-immunoprecipitated with Ago2 [53]; the function of this interaction has not been elucidated. A possible outcome could be the export of an RNA, such as smithRNAs, as a ribonuclear–protein complex through PNPase activity.

Thus, in the lack of a putative export mechanism, it is conceivable that PNPase participates in RNA export. For instance, it would be important to understand whether the PNPase complex can swap to achieve the same conformation facing the matrix, which could be achieved through immunochemical analysis with a matrix-targeted probe or atomic force microscope.

### (c) Mitochondrial permeability transition pore

The mPTP is a voltage-sensitive channel present in the IMM that has been found in yeast [54], animals [54] and plants [55]. It is generally activated by an increase in Ca^2+^ levels in the matrix or by oxidative stress [56]. The mPTP structure is formed or regulated by adenine nucleotide transferase (ANT), F1FO (F)-ATP synthase (F-ATPase) or its subunits, phosphate carrier and cyclophilin D, and can be paired with VDAC of the OMM [56].

Under oxidative stress or inflammatory conditions, under both transient and long-term opening, mPTP can leak mtDNA fragments to the cytoplasm [20,21]. In this case, VDAC oligomerization allows export through the OMM; VDACs can work both ways for mtDNA import–export [20,39]. In a study on *S. cerevisiae*, DNA could be imported through the IMM either using the mPTP or the individual ANT [39]. The possibility of mPTP leaking ncRNAs is also supported by the electrochemical gradient, specifically the ΔΨm component, that is found across the IMM [40]. Therefore, mPTP is a good candidate for RNA export, at least in some biologically relevant circumstances, and could be paired with VDACs for export through the OMM.

### (d) Exaptation of oxidative phosphorylation subunits for mitochondrial transport

Mitochondria of *Leishmania tropica* import all tRNAs from the nucleus [44]. This euglenozoan surprisingly ‘recycles’ nuclear-encoded subunits of OXPHOS complexes: an IMM multiprotein complex, called RNA import complex (RIC), is sufficient to induce tRNA import into artificial phospholipid vesicles [57]. A component of RIC, called RIC1, is an ATPase, the RNA-stimulated ATPase [58]. According to the mechanism proposed, tRNA binding to RIC leads to ATP hydrolysis on the matrix side, the exit of protons to the intermembrane space, and finally tRNA import [58]. Notably, RIC1 is structurally homologous to the α subunit of F1 ATP synthase [59,60].

RIC1 is not the only component of RIC, which is an OXPHOS subunit. RIC8A is homologous to CYBC17 and is produced by a single gene [61]. Homology was suggested for RIC6 to complex III iron–sulphur protein Rieske and for RIC9 to COX6, both encoded by single genes [62]. RIC5 is another protein that appears to be homologous to the single gene that encodes for COX4 but is not essential for tRNA import activity [62]. Moreover, knockdown experiments showed that RIC subunits 1, 4A, 6, 8A, 8B and 9 (subgroup R6) are necessary and sufficient; a minimal voltage-gated translocation pore composed of RIC4A, RIC6, and RIC9 has been named R3 [63]. The diameter of the pore is 10–20 nm, way larger than VDACs (2.5 nm [64]), TOM40 (2–2.5 nm [65]) and TIM23 (1.3−2.4 nm [66]). Results on *T. brucei* found that tRNA import is independent of Rieske (RIC6) protein, suggesting the potential absence of RIC in another species within the same Tripanosomatidae family [67], where tRNA import is firmly associated with ATOM activity [68].

The presence of divergent OXPHOS subunits, such as CYBC17 having an unexplained N-terminal tail, which has been associated with tRNA binding of RIC8A [61], has been recorded in euglenozoans [69]. Another notable example is DUI organisms; DUI species commonly show elongated cox1, atp8 and cox2 in one of the two mtDNA types. The elongation in cox2 can be owing to either an insertion (up to 4.8 kb in *Scrobicularia plana* (Semelidae) [70]) or a terminal extension (e.g. Unionidae [25]). Male cox2 (Mcox2) gene elongations have been found in many unionids, as well as in other DUI bivalves such as the mytilid *Arcuatula senhousia*, and although there is no direct confirmation, multiple lines of evidence point to functions associated with the DUI system [71]. The sequences coding for COX2, including the extensions, carry signatures of purifying selection, indicating maintenance of functionality, and the proteins are often predicted to have additional trans-membrane helices, which could allow the protein to be exposed at the mitochondrial surface or to the intermembrane space [70,72–74]. It has been hypothesized that such extensions serve a role in mitochondrial inheritance and, more specifically, as a mitochondrial tag for either degradation or differential segregation. A peak expression of Mcox2 was found in *Venustaconcha ellipsiformis* (Unionidae) shortly before fertilization while it has low, uniform expression in male somatic tissue; Mcox2 localizes on both the IMM and the OMM in sperm [74]. The dual role of Mcox2 in both the OXPHOS pathway and reproduction reflects what has been found for RIC subunits. The timing of Mcox2 peak expression could suggest a functional implication of RNA exit in sex determination through RNAi-mediated gene regulation.

The *cox2* gene can also be duplicated in either sex in DUI species, further supporting its role in sex determination, as one copy could evolve sex-specific functions. For example, the F-mtDNA of *R. philippinarum* shows a *cox2* duplication, while in *A. senhousia* the duplication is found in the M-mtDNA [75]. Still different is *Limecola balthica* (Tellinidae), where an insertion divides M*cox2* into two genes, which can possibly be trans-spliced into a single mRNA [33].

## 5. Concluding remarks

We have gathered several, independent clues about mechanisms that may lead to RNA export from the mitochondrial matrix to the cytoplasm, which could further elucidate the role of RNA-mediated MRRs in eukaryotic cells. We suggest that focusing on structures like the TIM22/TIM23 complexes, PNPase, mPTP, and possibly exapted OXPHOS subunits, similar to the RIC complex, will provide stimulating insights in the next future. A comparative approach is an advantage in such studies, which contrasts with the consideration that most studies on RNA-mediated MRR have been performed on mammals and yeast (table 1). Potential applications of the exit mechanisms lie in their manipulation to alter the cascade effects of the RNA activity; an example could be seen in ASncmtRNAs, which are already a focus of cancer research. This new knowledge will lead to a better understanding of the distribution and functions of regulatory RNAs of mitochondrial origin among metazoans; future research in this field will shed light on a neglected aspect of retrograde signalling that is ripe for future discoveries on the coevolution and co-adaptation of the two eukaryotic genomes.

## Data Availability

This article has no additional data.
